# Self-perceived barriers to healthcare access for patients with post COVID-19 condition

**DOI:** 10.1186/s12913-024-11488-w

**Published:** 2024-09-06

**Authors:** Iris M. Brus, Inge Spronk, Suzanne Polinder, Alfons G. M. Olde Loohuis, Peter Tieleman, Stella C. M. Heemskerk, Sara Biere-Rafi, Juanita A. Haagsma

**Affiliations:** 1 Department of Public Health, Erasmus MC, Erasmus University Medical Centre Rotterdam, Rotterdam, Netherlands; 2 C-support, ‘s Hertogenbosch, Netherlands

**Keywords:** Post COVID-19 condition, Long COVID, Barriers, Healthcare access, Access to care, Determinants

## Abstract

**Background:**

Many patients with post COVID-19 condition (PCC) require healthcare services. However, qualitative studies indicate that patients with PCC encounter many barriers to healthcare access. This cross-sectional study aimed to determine how many PCC patients report barriers to healthcare access and which barriers are reported, and to explore differences between subgroups.

**Methods:**

Data were collected via an online survey from 10,462 adult patients with a confirmed or suspected COVID-19 infection in the Netherlands, who experienced persisting symptoms ≥ 3 months after the initial infection. To study self-perceived barriers, a list of eleven possible barriers was used, covering multiple aspects of healthcare access. Differences between subgroups based on sociodemographic characteristics, medical characteristics, PCC symptoms (fatigue, dyspnoea, cognitive problems, anxiety and depression), and healthcare use (general practitioner, paramedical professional, medical specialist, occupational physician and mental health professional) were studied through multivariable multinomial (0 vs. 1 vs. > 1 barrier) and binomial regression analyses (for each individual barrier).

**Results:**

A total of 83.2% of respondents reported at least one barrier to healthcare access. Respondents reported a median of 2.0 (IQR = 3.0) barriers. The barriers “I didn’t know who to turn to for help” (50.9%) and “No one with the right knowledge/skills was available” (36.8%) were most frequently reported. Respondents with younger age, higher educational level, not hospitalized during acute COVID-19 infection, longer disease duration, who had more severe PCC symptoms, and who did not consult an occupational physician or paramedical professional, were more likely to report barriers. Analyses per barrier showed that women were more likely to report financial and help-seeking barriers, while men were more likely to report barriers related to availability of care. Hospitalized respondents were less likely to report barriers related to availability of care, but not less likely to report financial or help-seeking barriers.

**Conclusions:**

This study shows that the majority of patients with PCC experiences barriers to healthcare access. Particular attention should be paid to younger, non-hospitalized patients with a long disease duration and severe PCC symptoms. Efforts to remove barriers should focus not only on improving availability of care, but also on helping patients navigate care pathways.

**Supplementary Information:**

The online version contains supplementary material available at 10.1186/s12913-024-11488-w.

## Background

The long-term effects of COVID-19 are increasingly recognized as a major public health challenge on a global scale [1]. Since the onset of the worldwide pandemic in 2020, there have been over 770 million confirmed cases of COVID-19 and although most patients recover shortly after the acute infection, an estimated 4–12% experience persisting symptoms [2–4]. The World Health Organization refers to these persisting symptoms as 'post COVID-19 condition' (PCC), defined as the continuation or development of symptoms occurring three months after the initial infection and lasting for at least two months, without any other explanation [5]. These symptoms encompass a broad spectrum, including fatigue, shortness of breath and cognitive problems, and appear to affect patients with both a mild and severe acute disease course [3, 5].

Healthcare services for individuals with COVID-19 have rapidly been set up since the start of the pandemic, primarily for those with severe symptoms during the acute infection, through expanding the number of critical care beds, additional staffing and equipment, and temporary hospitals [6]. Less extensively, services have become available for those with PCC, primarily focusing on rehabilitation, with different care pathways being developed and studied [7, 8]. Support for PCC patients is urgently needed, as a substantial number of patients suffering from PCC require healthcare services due to their persisting symptoms. Previous research shows increased healthcare utilization of PCC patients in the years following infection [9–12]. Given the substantial number of individuals likely affected by PCC, this increased healthcare utilization places a large burden on healthcare systems.

The care for PCC poses several challenges. Although the body of research on PCC is rapidly increasing and several different hypotheses are being studied, the pathophysiology remains unknown [13]. In addition, due to the complexity of the condition and the wide range of symptoms, previous studies emphasized the need for multidisciplinary, integrative care, involving many different specialties [7, 14]. Moreover, as the degree of severity and the extent of functional limitations vary widely among those affected and as PCC might have a fluctuating or relapsing nature, adequate care likely requires tailoring to the needs of individual patients [7, 14]. In spite of these challenges and the remaining uncertainty regarding the effects of rehabilitation for PCC, current evidence on the effectiveness of rehabilitation services for these patients suggests that it has beneficial effects on symptoms, functional limitations and quality of life [7, 8, 15].

Ensuring that PCC patients have access to adequate care is crucial, as earlier studies in other patient populations concluded that experiencing barriers to healthcare access is negatively associated with health-related quality of life and other health outcomes [16–18]. Yet previous research showed that patients with PCC experience difficulties in finding adequate care, including being unable to access care, long waiting lists, not being taken seriously, receiving conflicting and inconsistent advice, and fragmented healthcare services, i.e. lack of coordination between healthcare providers and no overall assessment of the impact of PCC [19–25]. Although these studies highlight relevant problems with current PCC care, the vast majority were of a qualitative nature, conducted in relatively small study populations. Research in a large population of PCC patients is needed to clarify the extent of these problems regarding access to care. Furthermore, it remains unknown whether certain subgroups of PCC patients experience more barriers to access healthcare than others. Previous studies in patient populations with other chronic diseases showed that factors such as gender, educational level, presence of comorbidities and disease severity are associated with experiencing barriers to care [26].

In order to ensure adequate healthcare access for PCC patients and to tackle barriers experienced by these patients, this study aimed to determine how many PCC patients report barriers to healthcare access, which barriers they report, and to explore differences between subgroups based on sociodemographic characteristics, medical characteristics, the presence of PCC symptoms and healthcare use. Based on previous qualitative research, we hypothesize that barriers experienced by PCC patients are related to different aspects of the healthcare system, including the navigation through available services. In addition, we hypothesize that patients with a low educational level, who have not been hospitalized, who experience severe symptoms and who experience cognitive problems have a higher likelihood of reporting barriers to healthcare access than other patients.

## Methods

### Study design, data collection and participants

For this cross-sectional study, data were collected via an online survey from patients with PCC registered at C-support. C-support is a Dutch foundation, commissioned by the Ministry of Health, that informs, advises and supports patients who experience long-term complaints after a confirmed or suspected COVID-19 infection. Patients can self-register at C-support if they experience symptoms and/or functional limitations at least 3 months after a COVID-19 infection. To register, patients are asked to complete an online form with some personal information and contact details, after which they are contacted by C-support to assess the need and possibility for support. Between February 2022 and February 2023, a total of 19,249 patients of all ages who were part of the C-support PCC registry were invited via email to participate in the study. If patients did not complete the survey within three weeks, a reminder email was sent. The survey was available in Dutch, and respondents were able to complete the survey in steps by saving their answers and resuming the survey later. Inclusion criteria for the present study were: age ≥ 18 years when completing the survey, an infection date ≥ 3 months prior to completing the survey, no missing data on PCC symptoms, reporting to have needed healthcare services, and having responded to the survey question on healthcare access. All data used in this study were extracted from the survey.

## Measures

### Sociodemographic characteristics

The survey contained questions on sociodemographic characteristics, including age (in years), gender, educational level, and ethnicity. Age was categorized into six groups: 18–24, 25–34, 35–44, 45–54, 55–64, and 65 years and older. Gender contained the following categories: man, woman, other, and rather not disclose. Educational level was categorized into three groups according to the International Standard Classification of Education (ISCED): low, middle, and high [27]. For ethnicity, the response options consisted of a list of the most common ethnicity groups in the Netherlands, an open option, and the option 'rather not disclose'.

### Medical characteristics

Self-reported medical characteristics included month and year of initial COVID-19 infection, hospitalization during acute infection (yes/no), and the presence of comorbidity. Time since initial COVID-19 infection was calculated based on the number of months between initial infection and completing the survey, and was categorized into four groups: ≤ 6 months, 7–12 months, 13–18 months, and > 18 months. For comorbidity, the question consisted of a list of 14 chronic diseases (including asthma, COPD or chronic emphysema, inflammatory bowel disease, stroke, depressive disorder or anxiety disorder, serious heart or vascular problems, arthrosis, rheumatism, serious back problems, hypertension, cancer, diabetes, and thyroid abnormalities) and the options “other chronic disease” and “no chronic disease”. Respondents were categorized into two groups: no comorbidity and comorbidity [28].

### PCC symptoms

Several self-reported PCC symptoms were assessed in the survey, including fatigue, dyspnoea, cognitive problems, anxiety, and depression. These symptoms were selected based on available information on commonly reported symptoms by PCC patients, and symptoms for which a standardized instrument was available. Fatigue was measured using the subscale fatigue severity of the Checklist Individual Strength (CIS) [29]. This subscale consists of eight items on a 7-point Likert scale. Total scores range from 8 to 56, and a score of 35 or higher is indicative of severe fatigue [30]. Dyspnoea was measured using the Medical Research Council (MRC) Dyspnoea Scale [31]. This scale assesses the degree of functional disability due to dyspnoea and ranges from grade 1 to 5. To measure cognitive problems, we used an additional item, or “bolt-on”, cognition for the EQ-5D-5L, a generic instrument to measure health-related quality of life [32, 33]. Cognition was defined as “remembering, understanding, concentrating, thinking”. Respondents could select one of five response categories: “no problems”, “slight problems”, “moderate problems”, “severe problems” and “extreme problems”. Anxiety was measured using the GAD-2, the short version of the Generalized Anxiety Disorder 7-item questionnaire [34]. This version consists of two items assessing how often respondents were affected by each symptom during the last two weeks, with response categories ranging from 0 (“not at all”) to 3 (“nearly every day”). Total scores range from 0 to 6 and a score of 3 or higher indicates a possible generalized anxiety disorder. Depression was measured using the PHQ-2, the short version of the Patient Health Questionnaire 9-item [35], which also consists of two items with answers ranging from 0 (“not at all”) to 3 (“nearly every day”). A score of 3 or higher indicates a possible depressive disorder [36]. Although the instruments used to measure PCC symptoms have been validated in different patient population, they were not validated in PCC patients, due to the recent emergence of this condition.

### Healthcare use

To assess healthcare use, respondents were asked which healthcare providers they had consulted for their complaints since the initial COVID-19 infection. Based on input from healthcare professionals and PCC patients, a list of 19 conventional healthcare providers was compiled. For the analyses, five dichotomous variables were created to determine whether respondents had consulted (1) a general practitioner, (2) a paramedical professional (including physiotherapist, occupational therapist, dietician or nutritionist, speech therapist, manual therapist, Cesar therapist, or Mensendieck therapist), (3) a medical specialist (including pulmonologist, internal medicine specialist, cardiologist, neurologist, rehabilitations specialist, ENT specialist, psychiatrist, or sports medicine specialist), (4) an occupational physician, or (5) a mental health professional (including psychologist, psychotherapist, or general practice mental health worker). Contextual information about the functioning of the healthcare system in the Netherlands and specific information about available PCC care is provided in Additional File 2.

### Self-perceived barriers to healthcare access

To measure self-perceived barriers to healthcare, we used a list of 11 possible barriers (Table 2) based on a report from the Netherlands Institute for Health Services Research on the self-management of patients with chronic conditions [37]. The original list consisted of 14 barriers: two of the original barriers (“I couldn't find the specific help I wanted” and “I was not aware of the rules or procedures for asking for help”) were not included, as there was some overlap between barriers. In addition, the barriers “No one with the right knowledge was available” and “No one with the right skills was available” were merged into “No one with the right knowledge and/or skills was available”. The question was formulated as “Have you ever encountered one or more of the following problems when arranging healthcare services?”. Respondents could answer “Yes” or “No” for each barrier, in addition to the exclusive answer options “No, I have not encountered any of these problems” or “Not applicable, I did not need healthcare services”. Respondents could also select an option “Other, namely”. Respondents who did not need healthcare services or who only selected the option “Other” were excluded. Due to the limited number of characters available for respondents to elaborate on the option “Other” and the wide variety in provided answers, we were unable to use this information in the analyses.

### Data analyses

Descriptive statistics were performed for sociodemographic characteristics, medical characteristics, PCC symptoms, and healthcare use. The total number of experienced barriers was reported (median and interquartile range (IQR)), as well as the proportion of respondents who reported each barrier. In addition, Pearson correlation coefficients between each pair of barriers were reported. To determine whether sociodemographic characteristics, medical characteristics, PCC symptoms or healthcare use were associated with self-perceived barriers to healthcare access, logistic regression analyses were performed. As the proportion of patients from ethnic minority groups was very low, this variable was not included in the analyses due to the lack of statistical power. First, multinomial logistic regression analyses were done with the number of reported barriers as the dependent variable, categorized into three groups: 0 reported barriers (reference), 1 barrier and > 1 barrier. Multinomial logistic regression was chosen as cumulative odds ordinal logistic regression was not possible due to violation of the assumption of proportional odds. The number of barriers was categorized into three groups as we hypothesized that respondents reporting only one barrier might differ from respondents reporting > 1 barrier, and this categorization provided additional insight into the association between independent variables and the likelihood of reporting barriers. Subsequently, the different types of barriers patients experienced were studied. As correlation coefficients between individual barriers were relatively low, we decided not to categorize the barriers and did not perform a cluster analysis. Instead, each barrier was studied individually to determine the association with sociodemographic characteristics, medical characteristics, PCC symptoms and healthcare use. Thus, binomial logistic regression analyses were performed for each individual barrier (coded as “did not experience specific barrier” versus “experienced specific barrier”). For both the multinomial and binomial logistic regression analyses, a backward stepwise selection process was used to determine the independent variables included in the final model, removing variables with the largest *p*-value until all remaining variables had a statistically significant *p*-value (< 0.05). All independent variables were categorical, and the largest category (i.e. the category containing most respondents) was selected as the reference category. The assumption of multicollinearity was checked (variance inflation factor < 10 indicating no multicollinearity). Odds ratios (OR), 95% confidence intervals (95%-CI) and *p*-values were reported. All analyses were performed using IBM SPSS version 28.

## Results

A total of 19,249 patients were invited to participate, of whom 11,230 completed the survey (58.3%). Of those, 211 respondents (1.9%) were excluded from the analyses because they were younger than 18 years old when completing the survey, infection date was unknown, infection date was less than 3 months prior to completing the survey, or because data on PCC symptoms was missing. An additional 557 respondents (5.1%) were excluded because they had not needed healthcare services (*n* = 209), or because they had only responded ‘Other’ to the survey question on healthcare access (*n* = 348). Thus, 10,462 respondents (93.2%) were included in the analyses (Additional File 1, Fig. 1).

The median age of respondents was 48.0 years (IQR = 17.0), and the majority were women (76.0%) and had a high educational level (54.0%) (Table 1). Almost half of the respondents had a comorbidity (47.0%), and 8.0% was hospitalized during the acute COVID-19 infection. Time since infection ranged from 3 to 35 months, with 17.0% infected ≤ 6 months prior to completing the survey, and 30.4% infected > 18 months prior. Most respondents experienced slight to extreme cognitive problems (92.4%), had severe fatigue (89.5%), and experienced at least some functional impairment due to dyspnoea (67.2%). About one quarter of respondents had a possible depressive disorder (28.9%) or anxiety disorder (24.8%). Most respondents had consulted a general practitioner (95.4%), a paramedical professional (93.4%), an occupational physician (74.0%), or a medical specialist (61.8%) for their complaints, while fewer respondents had consulted a mental health professional (45.7%).

**Table 1:** Characteristics of total study population and respondents reporting 0, 1 and > 1 barrier

		Total population	0 barriers	1 barrier	>1 barrier	
		*N*=10,462	*N*=1,757 (16.7%)	*N*=2,024 (19.3%)	N=6,681 (63.9%)	
		*Median (IQR)*				*p*-value^g^
*Sociodemographic characteristics*	Age in years	48.0 (17.0)	52.0 (14.0)	50.0 (16.0)	46.0 (17.0)	
		*N (%)*				
	**Age in categories**					< 0.001
	18–24 years	271 (2.6)	18 (1.0)	47 (2.3)	206 (3.1)	
	25–34 years	1,465 (14.0)	115 (6.5)	198 (9.8)	1,152 (17.2)	
	35–44 years	2,318 (22.2)	295 (16.8)	415 (20.5)	1,608 (24.1)	
	45–54 years	3,325 (31.8)	584 (33.2)	660 (32.6)	2,081 (31.1)	
	55–64 years	2,625 (25.1)	631 (35.9)	594 (29.3)	1,400 (21.0)	
	65–88 years	458 (4.4)	114 (6.5)	110 (5.4)	234 (3.5)	
	**Gender**					0.223
	Men	2,485 (23.8)	442 (25.2)	490 (24.2)	1,553 (23.2)	
	Women	7,947 (76.0)	1,310 (74.6)	1,532 (75.7)	5,105 (76.4)	
	Other	21 (0.2)	4 (0.2)	2 (0.1)	15 (0.2)	
	Rather not disclose	9 (0.1)	1 (0.1)	-	8 (0.1)	
	**Educational level**					< 0.001
	Low	1,239 (11.8)	312 (17.8)	320 (15.8)	607 (9.1)	
	Middle	3,552 (34.0)	654 (37.2)	729 (36.0)	2,169 (32.5)	
	High	5,652 (54.0)	784 (44.6)	968 (47.8)	3,900 (58.4)	
	Unknown^a^	19 (0.2)	7 (0.4)	7 (0.3)	5 (0.1)	
*Medical characteristics*	**Comorbidity**					< 0.001
	Yes	4,919 (47.0)	874 (49.7)	992 (49.0)	3,053 (45.7)	
	No	5,543 (53.0)	883 (50.3)	1,032 (51.0)	3,628 (54.3)	
	**Hospital admission during acute COVID-19 infection**					< 0.001
	Yes	842 (8.0)	221 (12.6)	183 (9.0)	438 (6.6)	
	No	9,620 (92.0)	1,536 (87.4)	1,841 (91.0)	6,243 (93.4)	
	**Time since infection**					< 0.001
	≤ 6 months	1,774 (17.0)	398 (22.7)	370 (18.3)	1,006 (15.1)	
	7–12 months	2,754 (26.3)	548 (31.2)	576 (28.5)	1,630 (24.4)	
	13–18 months	2,751 (26.3)	452 (25.7)	551 (27.2)	1,748 (26.2)	
	> 18 months	3,183 (30.4)	359 (20.4)	527 (26.0)	2,297 (34.4)	
*PCC symptoms*	**Severe fatigue**^**b**^					< 0.001
	Yes	9,364 (89.5)	1,497 (85.2)	1,762 (87.1)	6,105 (91.4)	
	No	1,098 (10.5)	260 (14.8)	262 (12.9)	576 (8.6)	
	**Dyspnoea**^**c**^					< 0.001
	Grade 1	3,430 (32.8)	670 (38.1)	690 (34.1)	2,070 (31.0)	
	Grade 2	3,047 (29.1)	467 (26.6)	600 (29.6)	1,980 (29.6)	
	Grade 3	3,230 (30.9)	520 (29.6)	596 (29.4)	2,114 (31.6)	
	Grade 4	422 (4.0)	62 (3.5)	81 (4.0)	279 (4.2)	
	Grade 5	333 (3.2)	38 (2.2)	57 (2.8)	238 (3.6)	
	**Cognitive problems**^**d**^					< 0.001
	None	799 (7.6)	197 (11.2)	171 (8.4)	431 (6.5)	
	Slight	2791 (26.7)	525 (29.9)	588 (29.1)	1,678 (25.1)	
	Moderate	3975 (38.0)	657 (37.4)	765 (37.8)	2,553 (38.2)	
	Severe	2468 (23.6)	329 (18.7)	437 (21.6)	1,702 (25.5)	
	Extreme	429 (4.1)	49 (2.8)	63 (3.1)	317 (4.7)	
	**Possible anxiety disorder**^**e**^					< 0.001
	Yes	2,598 (24.8)	314 (17.9)	428 (21.1)	1,856 (27.8)	
	No	7,864 (75.2)	1,443 (82.1)	1,596 (78.9)	4,825 (72.2)	
	**Possible depressive disorder**^**f**^					< 0.001
	Yes	3,022 (28.9)	358 (20.4)	537 (26.5)	2,127 (31.8)	
	No	7,440 (71.1)	1,399 (79.6)	1,487 (73.5)	4,554 (68.2)	
*Healthcare use*	**General practitioner**					< 0.001
	Yes	9,982 (95.4)	1,667 (94.9)	1,872 (92.5)	6,443 (96.4)	
	No	480 (4.6)	90 (5.1)	152 (7.5)	238 (3.6)	
	**Paramedic**					< 0.001
	Yes	9,769 (93.4)	1,682 (95.7)	1,824 (90.1)	6,263 (93.7)	
	No	693 (6.6)	75 (4.3)	200 (9.9)	418 (6.3)	
	**Medical specialist**					< 0.001
	Yes	6,470 (61.8)	1,001 (57.0)	1,142 (56.4)	4,327 (64.8)	
	No	3,992 (38.2)	756 (43.0)	882 (43.6)	2,354 (35.2)	
	**Occupational physician**					< 0.001
	Yes	7,745 (74.0)	1,378 (78.4)	1,423 (70.3)	4,944 (74.0)	
	No	2,717 (26.0)	379 (21.6)	601 (29.7)	1,737 (26.0)	
	**Mental health professional**					
	Yes	4,777 (45.7)	678 (38.6)	793 (39.2)	3,306 (49.5)	< 0.001
	No	5,685 (54.3)	10,79 (61.4)	1,231 (60.8)	3,375 (50.5)	

^a^For educational level, respondents could choose an option ‘Other, namely’, and these open answers were recoded into existing categories. However, not all open answers could be classified, and thus educational level is unknown for 19 respondents

^b^Measured using the Checklist Individual Strength, short scale fatigue severity

^c^Measured using the Medical Research Council Dyspnea Scale

^d^Measured using an additional item cognition for the EQ-5D-5L

^e^Measured using the Generalized Anxiety Disorder 2-item questionnaire

^f^Measured using the Patient Health Questionnaire 2-item questionnaire

^g^Using chi-square test

Respondents who reported more barriers were slightly younger, had a higher educational level, less often had a comorbidity, were less often hospitalized and had a longer disease duration (*p* < 0.001) (Table 1). They had higher rates of fatigue, dyspnoea, cognitive problems, possible anxiety disorder and depressive disorder (*p* < 0.001). Healthcare use also significantly differed between the three groups. Respondents reporting 1 barrier were least likely to have consulted each type of healthcare provider, except a mental health professional. The largest percentage differences between groups were seen for medical specialist and mental health professional: a medical specialist was consulted by 56.4% of those reporting 1 barrier compared to 64.8% of those reporting > 1 barrier, and a mental health professional was consulted by 38.6% of those not reporting any barriers compared to 49.5% of those reporting > 1 barrier.

### Reported barriers

**Fig. 1:**
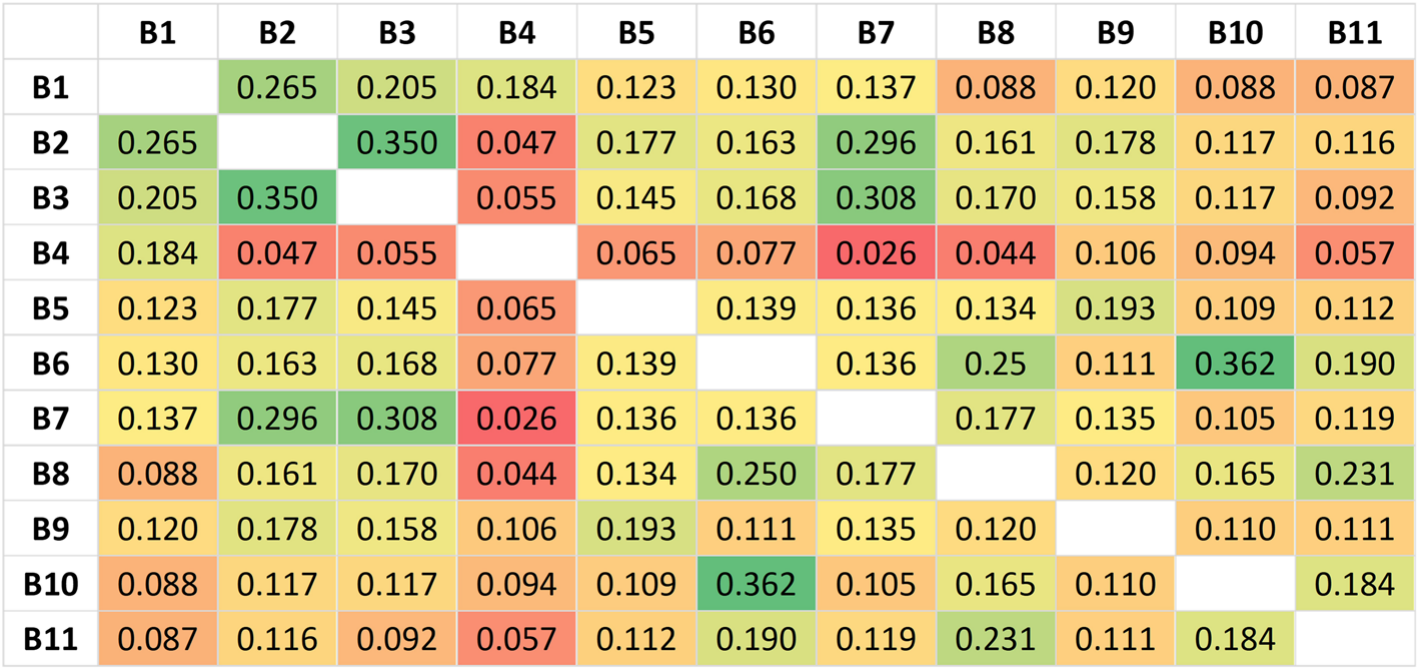
Heat map of correlation coefficients between each pair of barriers. See Table 2 for the corresponding barrier

A total of 83.2% of respondents reported at least one barrier to healthcare access; 19.3% reported 1 barrier, 63.9% reported > 1 barrier and 26.8% reported > 3 barriers (Additional File 1, Table 1). Respondents reported a median of 2.0 (IQR = 3.0) out of 11 barriers. The three most reported barriers were “I didn’t know who to turn to for help” (50.9%), “No one with the right knowledge and/or skills was available” (36.8%), and “The person I asked for help was unable to help me” (34.1%) (Table 2). The correlation between barriers is presented in Fig. 1, showing relatively low correlation coefficients ranging from 0.026 to 0.362. Barriers with the strongest correlation were “The help I sought was not reimbursed” and “The help or aid I wanted was too expensive” (0.362), followed by “No one with the right knowledge and/or skills was available” and “The person I asked for help was unable to help me” (0.350).

**Table 2:** Number and percentage of respondents who reported each barrier

	**Barrier**	**N**	**%**
B1	I didn’t know who to turn to for help	5,323	50.9
B2	No one with the right knowledge and/or skills was available	3,851	36.8
B3	The person I asked for help was unable to help me	3,567	34.1
B4	I felt uncomfortable asking for help, because I felt like a burden	3,239	31.0
B5	I had to wait a long time until help was available	2,708	25.9
B6	The help I sought was not reimbursed	2,424	23.2
B7	No help was available for my specific needs	1,437	13.7
B8	According to the care provider/organization, I was not eligible for help	1,366	13.1
B9	The person I asked for help didn’t have time	986	9.4
B10	The help or aid I wanted was too expensive	787	7.5
B11	It was difficult to apply for help due to complicated laws and regulations	534	5.1

### Differences between subgroups based on sociodemographic characteristics

#### Age

Multinomial logistic regression analyses showed that younger respondents had higher odds of reporting 1 and > 1 barrier compared to no barriers than older respondents (e.g. 45–54 years = reference; 18–24 years: OR_1 barrier_ = 2.006, *p* = 0.015 and OR_>1 barrier_ = 3.042, *p* < 0.001) (Table 3). Binomial logistic regression analyses showed the same pattern for each individual barrier: younger age was associated with higher odds of reporting each barrier (Additional File 1, Table 2A-D).

**Table 3:** Multivariable multinomial logistic regression analyses for number of reported barriers

		*Dependent variable category*						
	**1 barrier**				** > 1 barrier**			
*Independent variables*	**OR**	**95%-CI**		***p*-value**	**OR**	**95%-CI**		***p*-value**
		**Low**	**Upp**			**Low**	**Upp**	
**Gender**	N.S				N.S			
Women (ref)								
Men								
**Age in categories**								
18–24 years	2.006	1.143	3.523	.015	3.042	1.841	5.026	< .001
25–34 years	1.523	1.173	1.977	.002	2.570	2.062	3.202	< .001
35–44 years	1.265	1.047	1.529	.015	1.476	1.257	1.733	< .001
45–54 years (ref)	-	-	-	-	-	-	-	-
55–64 years	.807	.686	.949	.010	.608	.529	.698	< .001
65–88 years	.724	.532	.987	.041	.523	.399	.685	< .001
**Educational level**								
Low	.865	.713	1.049	.141	.439	.370	.521	< .001
Middle	.896	.775	1.037	.142	.643	.568	.728	< .001
High (ref)	-	-	-	-	-	-	-	-
**Comorbidity**	N.S				N.S			
No (ref)								
Yes								
**Hospital admission**								
No (ref)	-	-	-	-	-	-	-	-
Yes	.726	.581	.908	.005	.550	.455	.666	< .001
**Time since infection**								
≤ 6 months	.515	.415	.639	< .001	.301	.251	.362	< .001
7–12 months	.646	.535	.779	< .001	.397	.338	.466	< .001
13–18 months	.797	.661	.961	.017	.562	.479	.659	< .001
> 18 months (ref)	-	-	-	-	-	-	-	-
**Severe fatigue**								
No	.965	.787	1.183	.732	.730	.611	.873	< .001
Yes (ref)	-	-	-	-	-	-	-	-
**Dyspnoea**								
Grade 1 (ref)	-	-	-	-	-	-	-	-
Grade 2	1.247	1.055	1.473	.010	1.218	1.056	1.405	.007
Grade 3	1.139	.960	1.351	.135	1.177	1.019	1.360	.027
Grade 4	1.304	.907	1.874	.151	1.314	.961	1.797	.087
Grade 5	1.343	.864	2.085	.190	1.682	1.153	2.453	.007
**Cognition**								
None	.685	.536	.877	.003	.607	.493	.749	< .001
Slight	.977	.830	1.150	.779	.897	.780	1.031	.126
Moderate (ref)	-	-	-	-	-	-	-	-
Severe	1.114	.929	1.336	.245	1.177	1.008	1.373	.039
Extreme	.955	.642	1.419	.818	1.104	.794	1.534	.556
**Anxiety Disorder**								
No (ref)	-	-	-	-	-	-	-	-
Yes	1.018	.844	1.229	.848	1.300	1.109	1.523	.001
**Depression**								
No (ref)	-	-	-	-	-	-	-	-
Yes	1.344	1.125	1.606	.001	1.496	1.285	1.742	< .001
**General practitioner**								
No	1.368	1.033	1.811	.029	.776	.595	1.014	.063
Yes (ref)	-	-	-	-	-	-	-	-
**Paramedic**								
No	2.170	1.627	2.893	< .001	1.649	1.259	2.160	< .001
Yes (ref)	-	-	-	-	-	-	-	-
**Medical specialist**								
No	1.017	.879	1.178	.818	.809	.713	.917	< .001
Yes (ref)	-	-	-	-	-	-	-	-
**Occupational physician**								
No	1.485	1.258	1.751	< .001	1.477	1.278	1.707	< .001
Yes (ref)	-	-	-	-	-	-	-	-
**Mental health professional**								
No	1.036	.900	1.192	.624	.844	.750	.951	.005
Yes (ref)	-	-	-	-	-	-	-	-

A total of 10,413 respondents were included in regression analyses, due to missing data on gender or educational level (*n* = 49). The categorical dependent variable consisted of three groups: 0 (reference), 1 and > 1 barrier. N.S. = not significant (*p* > 0.05)

#### Gender

No statistically significant association was found between gender and reporting 1 and > 1 barrier compared to no barriers. However, analyses per barrier showed that some barriers were significantly more often experienced by women compared to men, namely: “I felt uncomfortable asking for help, because I felt like a burden” (OR = 0.707; p < 0.001), “The help or aid I wanted was too expensive” (OR = 0.707, *p* < 0.001), “The help I sought was not reimbursed (OR = 0.830, *p* = 0.002), and “According to the care provider/organization, I was not eligible for help” (OR = 0.830, *p* = 0.013). In contrast, the following barriers were significantly more often experienced by men: “No help was available for my specific needs” (OR = 1.445, *p* < 0.001), “The person I asked for help was unable to help me”(OR = 1.227, *p* < 0.001), “I didn’t know who to turn to for help” (OR = 1.171, *p* < 0.001), and “No one with the right knowledge and/or skills was available” (OR = 1.144, *p* = 0.009).

#### Educational level

Respondents with a low or middle educational level had lower odds of reporting > 1 barrier compared to no barriers than those with a high educational level (low educational level: OR_>1 barrier_ = 0.439, *p* < 0.001; middle educational level: OR_>1 barrier_ = 0.643, *p* < 0.001). Respondents with a lower educational level also had lower odds of reporting each individual barriers, except “The help or aid I wanted was too expensive” and “It was difficult to apply for help due to complicated laws and regulations”, for which no significant association was found.

### Differences between subgroups based on medical characteristics

#### Comorbidity

No association was found between comorbidity and reporting 1 and > 1 barrier compared to no barriers. However, analyses per barrier did show a significant association with two barriers. Those with comorbidity had lower odds of reporting the barriers “The help I sought was not reimbursed” (OR = 0.829, *p* < 0.001) and “No one with right knowledge and/or skills was available” (OR = 0.865, *p* = 0.001) than those without comorbidity.

#### Hospitalization during acute COVID-19 infection

Hospitalized respondents had lower odds of reporting 1 and > 1 barrier compared to no barriers than non-hospitalized respondents (OR_1 barrier_ = 0.726, *p* = 0.005; OR_>1 barrier_ = 0.550, *p* < 0.001). Analyses per barriers showed that hospital admission was also significantly associated with lower odds of reporting the following five barriers: “No one with right knowledge and/or skills was available” (OR = 0.622, *p* < 0.001), “The person I asked for help was unable to help me” (OR = 0.628, *p* < 0.001), “No help was available for my specific needs” (OR = 0.632, *p* < 0.001), “I had to wait a long time until help was available” (OR = 0.657, *p* < 0.001) and “I didn’t know who to turn to for help” (OR = 0.803, p = 0.004). However, hospitalized respondents had higher odds of reporting “It was difficult to apply for help due to complicated laws and regulations” (OR = 1.396, p = 0.021).

#### Time since infection

Respondents infected more recently had lower odds of reporting 1 and > 1 barrier compared to no barriers than those infected earlier (e.g. > 18 months = reference;  ≤ 6 months: OR_1 barrier_ = 0.515, *p* < 0.001 and OR_>1 barrier_ = 0.301, *p* < 0.001). Analyses per barrier showed the same pattern for all barriers, except for “The person I asked for help didn’t have time”, for which no significant association was found.

### Differences between subgroups based on PCC symptoms

#### Severe fatigue

Respondents without severe fatigue had lower odds of reporting > 1 barrier compared to no barriers than those who experienced severe fatigue (OR_>1 barrier_ = 0.730, p < 0.001). Analyses per barrier showed that those not experiencing severe fatigue also had lower odds of reporting the following individual barriers: “The help I sought was not reimbursed” (OR = 0.713, p < 0.001), “According to the care provider/organization, I was not eligible for help” (OR = 0.772, *p* = 0.026), “I had to wait a long time until help was available” (OR = 0.819, *p* = 0.025), and “I felt uncomfortable asking for help, because I felt like a burden” (OR = 0.811, *p* = 0.012).

#### Dyspnoea

Dyspnoea was significantly associated with reporting barriers: compared to respondents with grade 1, those with more severe dyspnoea had higher odds of reporting > 1 barrier (e.g. grade 1 = reference; grade 5: OR_>1 barrier_ = 1.682, *p* = 0.007), although no significant association was found between grade 4 and grade 1. Dyspnoea was also significantly associated with all individual barriers, except “I didn’t know who to turn to for help”, “The help I sought was not reimbursed” and “No help was available for my specific needs”.

#### Cognitive problems

Cognitive problems were also associated with reporting barriers, although the association was only statistically significant when comparing no problems to moderate problems (moderate = reference; OR_1 barrier_ = 0.685, *p* < 0.001 and OR_>1 barrier_ = 0.607, *p* < 0.001) and severe problems to moderate problems (OR_>1 barrier_ = 1.177, *p* < 0.001). Those experiencing more severe cognitive problems also had higher odds of reporting individual barriers, except for “The person I asked for help didn’t have time”.

#### Anxiety and depression

Respondents with a possible anxiety disorder had higher odds of reporting > 1 barrier compared to no barriers than those without a possible anxiety disorder (OR_>1 barrier_ = 1.300, *p* = 0.001). Similarly, respondents with a possible depressive disorder had higher odds of reporting 1 and > 1 barrier compared to no barriers than those without a possible depressive disorder (OR_1 barrier_ = 1.344, *p* = 0.001; OR_>1 barrier_ = 1.496, *p* < 0.001). In addition, those with possible anxiety disorder had higher odds of reporting 7 out of 11 individual barriers and those who had a possible depressive disorder had higher odds of reporting 5 out of 11 barriers. Only “According to the care provider/organization, I was not eligible for help” was not significantly associated with either possible anxiety disorder or possible depressive disorder.

### Differences between subgroups based on healthcare use

#### General practitioner

For general practitioner, there was a significant association when comparing those reporting 1 barrier to those not reporting any barriers: respondents who had not consulted a general practitioner had higher odds of reporting 1 barrier compared to no barriers than those who had consulted a general practitioner (OR_1 barrier_ = 1.368, *p* = 0.029). In contrast, analyses per barrier showed that those not having consulted a general practitioner had lower odds of reporting the following five barriers: “The person I asked for help was unable to help me” (OR = 0.488, *p* < 0.001), “No help was available for my specific needs” (OR = 0.626, *p* = 0.008), “No one with the right knowledge and/or skills was available” (OR = 0.682, *p* < 0.001), “The help I sought was not reimbursed” (OR = 0.682, *p* = 0.005), and “I felt uncomfortable asking for help, because I felt like a burden” (OR = 0.755, *p* = 0.012).

#### Paramedical professional

Respondents who had not consulted a paramedical professional had higher odds of reporting 1 and > 1 barrier compared to no barriers than those who had consulted a paramedical professional (OR_1 barrier_ = 2.170, *p* < 0.001; OR_>1 barrier_ = 1.649, *p* < 0.001). Those not having consulted a paramedical professional also had higher odds of reporting the barriers “I felt uncomfortable asking for help, because I felt like a burden” (OR = 1.433, *p* < 0.001) and “No help was available for my specific needs” (OR = 1.296, *p* = 0.028). However, they had lower odds of reporting the barriers “No one with the right knowledge and/or skills was available” (OR = 0.657, *p* < 0.001) and “The help I sought was not reimbursed” (OR = 0.723, *p* = 0.004).

#### Medical specialist

Respondents who had not consulted a medical specialist had lower odds of reporting > 1 barrier compared to no barriers than those who had consulted a medical specialist (OR_>1 barrier_ = 0.809, *p* < 0.001). Those who had not consulted a medical specialist also had lower odds of reporting 7 out of the 11 total barriers (ORs ranging from 0.550–0.806), but had higher odds of reporting the barrier “I felt uncomfortable asking for help, because I felt like a burden” (OR = 1.129, *p* = 0.014).

#### Occupational physician

Respondents who had not consulted an occupational physician had higher odds of reporting 1 and > 1 barrier compared to no barriers than those who had consulted an occupational physician (OR_1 barrier_ = 1.485, p < 0.001; OR_>1 barrier_ = 1.477, *p* < 0.001). Those not having consulted an occupational physician also had higher odds of reporting 7 individual barriers (ORs ranging from 1.191–1.550), but had lower odds of reporting the barrier “I had to wait a long time until help was available” (OR = 0.874, *p* = 0.020).

#### Mental health professional

Respondents who had not consulted a mental health professional had lower odds or reporting > 1 barrier compared to no barriers than those who had consulted a mental health professional (OR_>1 barrier_ = 0.844, *p* = 0.005). Those who had not consulted a mental health professional also had lower odds of reporting 8 individual barriers (ORs ranging from 0.595–0.877).

## Discussion

This study determined the extent to which PCC patients report barriers, which barriers they report, and explored differences between subgroups. We found that the majority of respondents experienced at least one barrier to healthcare access, with a median of 2 out of 11 barriers. The barriers most often reported were “I didn’t know who to turn to for help”, “No one with the right knowledge and/or skills was available” and “The person I asked for help was unable to help me”. The association between several independent variables and the number of reported barriers, as well as the types of barriers was studied. As correlations between barriers were relatively low, these analyses were performed for each barrier individually in order to stay as close as possible to the original data. Nevertheless, some barriers appeared to cover the same aspect of healthcare access, which was reflected in the pattern of associations with independent variables. These aspects of healthcare access include: financial barriers (“The help I sought was not reimbursed”, “The help or aid I wanted was too expensive” and “According to the care provider/organization, I was not eligible for help”), availability of care (“No one with the right knowledge and/or skills was available”, “The person I asked for help was unable to help me” and “No help was available for my specific needs”), and timeliness of care (“I had to wait a long time until help was available” and “The person I asked for help didn’t have time”), which is the terminology that will be used throughout the discussion. We found that respondents with lower age, higher educational level, who were not hospitalized during the acute COVID-19 infection, who had a longer disease duration, who had more severe PCC symptoms, and who had not consulted a paramedical professional or occupational physician had significantly higher odds of reporting 1 and > 1 barrier compared to no barriers to healthcare access. Analyses per barrier showed that women had higher odds of reporting financial barriers as well as feeling uncomfortable asking for help, while men had higher odds of reporting barriers related to availability of care. In addition, hospitalized respondents had lower odds of reporting barriers related to availability of care compared to non-hospitalized respondents.

### Reported barriers

The proportion of patients in our study population that reported at least one barrier to healthcare access was high: over 80% experienced at least one barrier, with over 25% reporting four barriers or more. In comparison, previous research on barriers to healthcare utilization among patients with Myalgic Encephalomyelitis/Chronic Fatigue Syndrome (ME/CFS), a similar disabling condition, found that 55% reported at least one barrier [38]. A recent study by Karpman et al. among adult PCC patients in the United States corroborates the multitude of barriers to accessing healthcare services experienced by this patient population: they concluded that PCC patients were more likely to report unmet healthcare needs compared to those with COVID-19 diagnosis but without PCC and those who tested negative for COVID-19 [24]. Their findings showed that unmet healthcare needs among PCC patients were attributable to challenges including costs of care, finding a healthcare professional accepting new patients, and getting a timely appointment. The proportion of PCC patients reporting financial barriers and barriers related to timeliness of care in this earlier study was similar to our results (financial: 27.0% in study Karpman vs. 8–23% in our study depending on barrier; timeliness: 22% vs. 9–26%). However, the most common barriers in our study were different: our respondents most frequently reported that they did not know where to go for help and that there was lack of availability of care, which were not investigated in the study by Karpman et al. Nevertheless, studies in other patient populations, including chronic disease patients, confirm that the availability of services is one of the most commonly cited barriers to healthcare access [38, 39].

Although we cannot fully elucidate the underlying causes of the reported barriers to healthcare access, these frequently reported barriers point towards several different problems with current PCC care. It appears that there is a lack of knowledge among healthcare providers consulted by PCC patients, resulting in patients receiving inadequate support, which has also been reported by earlier qualitative studies [19, 21, 25]. As PCC is a relatively new condition with, at the moment, an unknown pathophysiology, uncertain prognosis, and no curative treatment, the lack of knowledge and adequate support from healthcare providers is not surprising [13]. Nevertheless, this finding emphasizes the need to provide healthcare providers with clear and regularly updated clinical guidelines and to provide training and education to those involved in PCC care. Clearly, further development of the knowledge base, and continued funding for studies investigating the pathophysiology, prognosis and possible treatment options is also a priority in order to organize adequate care. In addition, the fact that over half of respondents report the barrier “I didn’t know who to turn to for help” highlights the need for easier navigation through health services and clear points of contact for patients. Previous qualitative studies similarly emphasized the importance of coordination and continuity of care to improve healthcare access, especially given the multifaceted nature of the condition, often requiring the involvement of many specialities [20, 22]. One of the suggested solutions is assigning the responsibility for care coordination to one designated clinician to ensure continuity of care for these patients [22].

The problems and solutions mentioned in the previous paragraph are all related to factors on a healthcare system-level. However, factors on a personal level, such as having insufficient skills to seek healthcare services (e.g. lacking health literacy), are also known to affect access to healthcare. A review on barriers to healthcare access for patients with Parkinson’s disease, a similarly complex condition involving many different healthcare disciplines, showed that barriers occur at both a person- and health system-level [40]. The authors of this study primarily emphasized the need to overcome person-level barriers, as they concluded that there is a lack of attention for these types of barriers. However, as PCC is a new condition with a rapidly increasing body of research, and as care pathways are still under development, it appears that efforts to improve access to PCC care should primarily focus on resolving barriers related health system-level factors, while paying attention to personal-level factors.

### Association with sociodemographic characteristics

Our findings show that several factors are associated with the number and type of barriers that PCC patients report. Lower age was associated with reporting more barriers, which is in line with previous research among patients with chronic diseases [41, 42]. Possible explanations for these age differences are a lack of experience in navigating health services or different expectations from these services [43]. Interestingly, although previous studies among patients with ME/CFS and other chronic diseases concluded that women are more likely to report barriers than men, we found no association between gender and the likelihood of reporting barriers [38, 41]. However, analyses per barrier showed that women and men reported different barriers, with women more often reporting financial barriers, which is in line with earlier research among patients with cardiovascular-related chronic diseases [44]. In contrast, men more often experienced barriers related to availability of care and knowing who to turn to for help.

In addition, our findings indicate that those with a high educational level report more barriers to healthcare access compared to those with a lower educational level. These results appear to be in contrast with previous studies that suggest either no association or an inverse association [38, 45]. We hypothesize that the association found in our study is at least partially due to the selection of participating PCC patients caused by the sampling method. All patients invited for this study self-registered in the C-support PCC registry. Due to the online self-registration process, highly educated respondents are likely overrepresented in our study sample, and low educated patients who experienced barriers might have been less likely to register compared to highly educated respondents who experience barriers. Another explanation for these surprising results could be that highly educated patients have higher levels of health literacy, and are possibly more familiar with ongoing research, current treatment and management options. Thus, they might be more aware of what is lacking in currently available PCC care, leading to highly educated patients reporting more barriers.

### Association with medical characteristics

Hospitalization during the acute COVID-19 infection was associated with reporting barriers to healthcare access, as our results showed that hospitalized respondents are less likely to report 1 and > 1 barrier than non-hospitalized respondents. Previous qualitative studies among PCC patients corroborate this finding, as they reported a lack of guidance for non-hospitalized patients [21]. Analyses per barrier showed that hospitalized patients have lower odds of reporting barriers mainly related to availability of care. A possible explanation for these findings is that hospitalized patients might receive rehabilitation or follow-up consultations after discharge from the hospital, thus having easier access to support for long-term complaints [8]. In addition, healthcare providers might be more aware of the possibility of long-term complaints for this patient group compared to non-hospitalized patients who experienced a mild acute disease course.

Besides hospitalization, disease duration was also associated with barriers to healthcare access, as those with a longer disease duration were more likely to report 1 and > 1 barrier. This might be due to the limited availability of healthcare services during the earlier phases of the pandemic. Half of our study population was infected in 2020, and during this phase, the healthcare system struggled to handle the large influx of patients with acute COVID-19, services for PCC were still in the process of being set-up, and knowledge of healthcare providers on this new condition was very limited [20, 46]. Aside from the pandemic phase, patients may experience more barriers the longer their symptoms last. As their symptoms and functional limitations continue to impact their daily life, and they possibly experience growing frustration with the available care, patients might be more likely to report barriers.

### Association with PCC symptoms

Furthermore, our findings showed that PCC patients who experienced severe fatigue, dyspnoea, cognitive problems, a possible anxiety disorder, or a possible depressive disorder, were more likely to experience 1 and > 1 barrier than those who do not experience these symptoms or who experience less severe symptoms. These results indicate that those with a more complex manifestation of PCC, i.e. more symptoms or more severe symptoms, are more likely to experience barriers to healthcare access. A previous systematic review similarly suggested that factors such as disease severity and reduced health status are associated with experiencing barriers to receiving optimal care among individuals with chronic diseases [26]. Due to the cross-sectional design of this study, the direction of the association between PCC symptoms and barriers to healthcare access remains unclear. Although earlier research found that experiencing barriers to healthcare access has a negative impact on a multitude of health outcomes, having more severe PCC symptoms could also increase the likelihood of reporting barriers [18]. Future research with a longitudinal design could elucidate the impact of barriers to accessing PCC care on health outcomes.

### Association with healthcare use

The association between healthcare use and barriers to access differed between the different types of healthcare providers. Respondents who had not consulted an occupational physician or paramedical professional were more likely to report 1 and > 1 barrier than those who had consulted these healthcare providers. In contrast, the inverse association was observed for medical specialists and mental health professionals: those who had consulted these providers actually more often reported barriers. For general practitioner, the association was unclear. Although these results seem somewhat conflicting, it does show that it is not the lack of access to a medical specialist that leads to barriers, as suggested in a previous study [22]. In addition, these findings seem to indicate that consulting a paramedical professional or occupational physician might lead to reporting less barriers. However, this does not appear to be the case for each individual barrier, so that conclusion should be interpreted with caution. When interpreting these results, it is also important to take into account that the timeline of healthcare use was not specified in the survey. Thus, whether respondents experienced barriers before or after they consulted a healthcare provider is unknown.

### Strengths and limitations

The strengths of this study include the large cohort of PCC patients and the broad range of possible barriers that was studied, which covered multiple aspects of healthcare access. In addition, by looking at a variety of factors that could influence perceived access to care simultaneously, we provide a clear overview of subgroups that have a high risk of reporting suboptimal healthcare access. Furthermore, the response rate of 58% was quite high, particularly considering the severity of symptoms reported by respondents.

However, this study also has several limitations. The primary limitation concerns the sampling method: the study population consisted of patients who self-registered at a PCC registry, meaning that respondents might not be representative of all PCC patients in the Netherlands. Respondents appear to have quite severe symptoms and we hypothesize that patients in our study population have a higher likelihood of reporting barriers to healthcare access compared to the average PCC patient. Thus, the high proportion of patient reporting barriers is possibly an overestimation. Furthermore, these patients might have higher health literacy than the average patient, as they were aware of the existence of C-support and registered themselves at this foundation in order to receive support. In line with this assumption, patients with a high educational level appear to be overrepresented in our sample. Nevertheless, we believe that our findings provide valuable insight into the most important barriers experienced by PCC patients and those who are most at risk for experiencing barriers. Second, it is important to mention that self-perceived barriers do not directly translate to actual healthcare access, as a higher likelihood of reporting barriers could also be attributable to factors other than poor access. For example, a previous study suggested that patients who perceive barriers may be more sensitive to unmet care needs as a result of more engagement in their care or higher degrees of health literacy [44]. Third, the survey question on healthcare access was broadly formulated, meaning that respondents could have interpreted the question as referring to general barriers to healthcare access, instead of barriers specifically pertaining to PCC care. However, the purpose of the study was clearly stated multiple times in the invitation, title and the survey itself. Fourth, although we included multiple sociodemographic and medical characteristics, as well as several ‘core’ symptoms of PCC, the list of possible determinants was not exhaustive. For example, previous studies found that patients that are part of an ethnic minority experience more barriers, as well as different barriers, compared to other patients [19]. However, as only a very small proportion of respondents in this study belonged to an ethnic minority, we were unable to investigate the association between ethnicity and self-perceived barriers to healthcare access. The lack of representation of ethnic minorities in our study could have led to an underestimation of specific barriers that are more often experienced by ethnic minorities. For example, these patients could experience more financial barriers, be less likely to ask for help due to cultural differences or have more difficulty communicating with healthcare providers. Thus, it is important to take this into account in future studies to provide more generalizable results and to determine the influence of ethnicity on possible disparities in healthcare access. Future studies should specifically target minority groups and possibly use a different sampling method (e.g. using patient records) to reach these respondents. Future studies should also further look into the impact of socio-economic status on healthcare access, as our unexpected findings regarding this association might be due to the sampling method, selection bias and/or non-response bias. Additionally, while we examined multiple common PCC symptoms, other symptoms such as post-exertional malaise (PEM), postural orthostatic tachycardia syndrome (POTS), and headache were not included in the survey. As recent research has shown that these symptoms, among others, are frequently reported and possibly pose challenges in receiving adequate care, a more extensive list of core symptoms should be included in future research [47–49]. Fifth, although we used validated questionnaires for most symptoms, cognitive problems were measured using a single item with five response options comparable to the EQ-5D-5L items. However, this single cognition item is not an officially validated instrument. Sixth, due to the cross-sectional nature of this study, it is unclear whether symptoms were already present before the COVID-19 infection; these symptoms are not necessarily due to PCC. Lastly, the data collection period spans over a year, during which changes occurred regarding public health measures that were in place, availability of healthcare services and awareness of PCC, which have not been accounted for in our analyses.

## Conclusion

The findings of this study show that many PCC patients experience barriers to healthcare access, with most of them having difficulty finding adequate support within the established healthcare facilities. The number and variety of barriers reported by patients highlights the complexity of organizing adequate care for this new and still relatively unknown condition. Nevertheless, addressing the obstacles that patients encounter when trying to access healthcare services is crucial, as PCC has a substantial impact on both patients and society, and suboptimal access to care could contribute to the persistence of long-term complaints. Efforts to improve healthcare access for this patient population should not only focus on the availability of healthcare services, but also on helping patients navigate care pathways, removing help-seeking barriers (e.g. feeling uncomfortable asking for help), and financial barriers. Creating national care paths for PCC patients with detailed guidelines about when to involve which professionals could provide both healthcare professionals and patients with clarity about treatment and support options. In addition, more patient education about the available care for PCC, government regulations and ongoing developments might also help patients navigate healthcare services, for example via patient information websites such as the Dutch Thuisarts.nl. A specific focus should be on providing easily accessible information for those with lower health literacy, low educational level and ethnic minority groups. Our study shows that sociodemographic characteristics, medical characteristics, and PCC symptom severity should be taken into account when addressing barriers, as these factors influence the number and type of barriers patients experience. Particular attention should be paid to younger, non-hospitalized patients with a long disease duration and severe PCC symptoms. We therefore recommend not only to increase awareness of the barriers experienced by PCC patients, but also educate key professionals in PCC care (e.g., general practitioner, physiotherapist, occupational physician, general practice mental health worker) on patient subgroups that have a higher likelihood of experiencing barriers. Additional research is needed to clarify the effect of factors such socioeconomic status and ethnicity, and to investigate potential measures to improve access to care for PCC patients.

## Supplementary Information

Supplementary Material 1.

Supplementary Material 2.

## Data Availability

The dataset supporting the conclusions of the current study is available for researchers who meet the criteria for access to data upon request which can be applied at the Data Access Committee of C-support.

## Abbreviations

CIS: Checklist Individual Strength
GAD-2: Generalized Anxiety Disorder 2-item questionnaire
IQR: Interquartile range
ME/CFS: Myalgic Encephalomyelitis/Chronic Fatigue Syndrome
MRC: Medical Research Council Dyspnoea Scale
OR: Odds ratio
PCC: Post COVID-19 condition
PEM: Post-exertional malaise
PHQ-2: Patient Health Questionnaire 2-item
POTS: Postural orthostatic tachycardia syndrome
95%-CI: 95% Confidence interval
