# Supplementary material for: Self-perceived barriers to healthcare access for patients with post COVID-19 condition
Source: BMC Health Serv Res. 2024 Sep 6;24:1035. doi: 10.1186/s12913-024-11488-w (PMC11378429; doi:10.1186/s12913-024-11488-w)
Supplement: Supplementary file 1 — Supplementary Material 1. [file 12913_2024_11488_MOESM1_ESM.docx]

# **Additional File 1**

**Figure 1. Flowchart of participant selection.**

**Total responses in dataset**

n=11,230

**Do meet inclusion criteria**

n=11,019

**Included in analyses**

n=10,462

**Healthcare access not relevant**

Healthcare access not applicable: n=209

Only selected option “other”: n=348

**Do not meet inclusion criteria**

<18 years old: n=85

Month infection unknown: n=10

Month infection <3 months ago: n=115

Data MRC missing: n=1

**Table 1. Number, proportion and cumulative proportion of respondents who reported barriers.**

| Total number of reported barriers | N | % | Cum. % |
| --- | --- | --- | --- |
| 0 | 1,757 | 16.8 | 16.8 |
| 1 | 2,024 | 19.3 | 36.1 |
| 2 | 2,114 | 20.2 | 56.3 |
| 3 | 1,761 | 16.8 | 73.2 |
| 4 | 1,146 | 11.0 | 84.1 |
| 5 | 753 | 7.2 | 91.3 |
| 6 | 411 | 3.9 | 95.3 |
| 7 | 262 | 2.5 | 97.8 |
| 8 | 127 | 1.2 | 99.0 |
| 9 | 61 | 0.6 | 99.6 |
| 10 | 33 | 0.3 | 99.9 |
| 11 | 13 | 0.1 | 100.0 |

**Table 2-A. Multivariable binomial logistic regression analyses per barrier for barriers 1-3.**

|  | Barrier 1 | | | | Barrier 2 | | | | Barrier 3 | | | |
| --- | --- | --- | --- | --- | --- | --- | --- | --- | --- | --- | --- | --- |
|  | **OR** | **95%-CI** | | **p-value** | **OR** | **95%-CI** | | **p-value** | **OR** | **95%-CI** | | **p-value** |
|  |  | **Low.** | **Up.** |  |  | **Low.** | **Up.** |  |  | **Low.** | **Up.** |  |
| Gender |  |  |  |  |  |  |  |  |  |  |  |  |
| Women (ref) | - | - | - | - | - | - | - | - | - | - | - | - |
| Men | 1.171 | 1.065 | 1.288 | .001 | 1.144 | 1.035 | 1.265 | .009 | 1.227 | 1.109 | 1.356 | <.001 |
| Age in categories |  |  |  |  |  |  |  |  |  |  |  |  |
| 18-24 years | 1.493 | 1.149 | 1.940 | .003 | 1.362 | 1.040 | 1.783 | .025 | 1.770 | 1.360 | 2.304 | <.001 |
| 25-34 years | 1.724 | 1.512 | 1.966 | <.001 | 1.520 | 1.329 | 1.737 | <.001 | 1.554 | 1.362 | 1.774 | <.001 |
| 35-44 years | 1.090 | .978 | 1.215 | .119 | 1.209 | 1.077 | 1.356 | .001 | 1.160 | 1.034 | 1.302 | .011 |
| 45-54 years (ref) | - | - | - | - | - | - | - | - | - | - | - | - |
| 55-64 years | .713 | .641 | .792 | <.001 | .731 | .651 | .821 | <.001 | .770 | .685 | .865 | <.001 |
| 65-88 years | .657 | .532 | .811 | <.001 | .818 | .652 | 1.026 | .082 | .631 | .498 | .801 | <.001 |
| Educational level |  |  |  |  |  |  |  |  |  |  |  |  |
| Low | .733 | .643 | .837 | <.001 | .513 | .442 | .594 | <.001 | .474 | .406 | .552 | <.001 |
| Middle | .808 | .740 | .882 | <.001 | .633 | .576 | .695 | <.001 | .664 | .604 | .729 | <.001 |
| High (ref) | - | - | - | - | - | - | - | - | - | - | - | - |
| Comorbidity | N.S. |  |  |  |  |  |  |  | N.S. |  |  |  |
| No (ref) |  |  |  |  | - | - | - | - |  |  |  |  |
| Yes |  |  |  |  | .865 | .793 | .944 | .001 |  |  |  |  |
| Hospital admission  during acute  COVID-19 infection |  |  |  |  |  |  |  |  |  |  |  |  |
| No (ref) | - | - | - | - | - | - | - | - | - | - | - | - |
| Yes | .803 | .691 | .932 | .004 | .622 | .526 | .736 | <.001 | .628 | .529 | .746 | <.001 |
| Time since infection |  |  |  |  |  |  |  |  |  |  |  |  |
| ≤6 months | .641 | .568 | .723 | <.001 | .338 | .293 | .390 | <.001 | .508 | .442 | .584 | <.001 |
| 7-12 months | .726 | .653 | .807 | <.001 | .440 | .392 | .494 | <.001 | .592 | .527 | .665 | <.001 |
| 13-18 months | .864 | .778 | .960 | .006 | .577 | .518 | .644 | <.001 | .798 | .716 | .891 | <.001 |
| >18 months (ref) | - | - | - | - | - | - | - | - | - | - | - | - |
| Severe fatigue | N.S. |  |  |  | N.S. |  |  |  | N.S. |  |  |  |
| No |  |  |  |  |  |  |  |  |  |  |  |  |
| Yes (ref) |  |  |  |  |  |  |  |  |  |  |  |  |
| Dyspnoea | N.S. |  |  |  |  |  |  |  |  |  |  |  |
| Grade 1 (ref) |  |  |  |  | - | - | - | - | - | - | - | - |
| Grade 2 |  |  |  |  | 1.003 | .901 | 1.117 | .959 | 1.088 | .977 | 1.213 | .125 |
| Grade 3 |  |  |  |  | .992 | .889 | 1.106 | .882 | 1.118 | 1.002 | 1.248 | .046 |
| Grade 4 |  |  |  |  | 1.124 | .899 | 1.405 | .304 | 1.083 | .864 | 1.358 | .490 |
| Grade 5 |  |  |  |  | 1.376 | 1.076 | 1.759 | .011 | 1.581 | 1.237 | 2.019 | <.001 |
| Cognitive problems |  |  |  |  |  |  |  |  |  |  |  |  |
| None | .585 | .497 | .688 | <.001 | .917 | .772 | 1.089 | .322 | 1.005 | .846 | 1.194 | .953 |
| Slight | .952 | .861 | 1.053 | .337 | 1.005 | .902 | 1.119 | .932 | .980 | .879 | 1.092 | .711 |
| Moderate (ref) | - | - | - | - | - | - | - | - | - | - | - | - |
| Severe | 1.075 | .969 | 1.193 | .172 | 1.168 | 1.047 | 1.304 | .005 | 1.186 | 1.063 | 1.323 | .002 |
| Extreme | 1.101 | .893 | 1.357 | .367 | 1.446 | 1.167 | 1.790 | <.001 | 1.314 | 1.062 | 1.626 | .012 |
| Likely anxiety  disorder |  |  |  |  |  |  |  |  | N.S. |  |  |  |
| No (ref) | - | - | - | - | - | - | - | - |  |  |  |  |
| Yes | 1.397 | 1.257 | 1.552 | <.001 | 1.196 | 1.081 | 1.323 | <.001 |  |  |  |  |
| Likely major  depressive disorder |  |  |  |  | N.S. |  |  |  |  |  |  |  |
| No (ref) | - | - | - | - |  |  |  |  | - | - | - | - |
| Yes | 1.172 | 1.059 | 1.296 | .002 |  |  |  |  | 1.174 | 1.066 | 1.293 | .001 |
| General practitioner | N.S. |  |  |  | - | - | - | - | - | - | - | - |
| No |  |  |  |  | .682 | .546 | .851 | <.001 | .488 | .383 | .621 | <.001 |
| Yes (ref) |  |  |  |  |  |  |  |  |  |  |  |  |
| Paramedic | N.S. |  |  |  | - | - | - | - | N.S. |  |  |  |
| No |  |  |  |  | .657 | .597 | .724 | <.001 |  |  |  |  |
| Yes (ref) |  |  |  |  |  |  |  |  |  |  |  |  |
| Medical specialist | N.S. |  |  |  | N.S. |  |  |  | - | - | - | - |
| No |  |  |  |  |  |  |  |  | .701 | .637 | .772 | <.001 |
| Yes (ref) |  |  |  |  |  |  |  |  |  |  |  |  |
| Occupational physician |  |  |  |  |  |  |  |  |  |  |  |  |
| No | 1.191 | 1.083 | 1.309 | <.001 | 1.129 | 1.020 | 1.249 | .019 | 1.128 | 1.020 | 1.248 | .019 |
| Yes (ref) | - | - | - | - | - | - | - | - | - | - | - | - |
| Mental health  professional | N.S. |  |  |  |  |  |  |  | N.S. |  |  |  |
| No |  |  |  |  | .869 | .795 | .949 | .002 |  |  |  |  |
| Yes (ref) |  |  |  |  | - | - | - | - |  |  |  |  |

A total of 10,413 respondents were included in regression analyses, due to missing data on gender or educational level (n=49). N.S.= not significant (p>0.05).

Barrier 1: I didn’t know who to turn to for help.

Barrier 2: No one with the right knowledge and/or skills was available.

Barrier 3: The person I asked for help was unable to help me.

**Table 2-B. Multivariable binomial logistic regression analyses per barrier for barriers 4-6.**

|  | Barrier 4 | | | | Barrier 5 | | | | Barrier 6 | | | |
| --- | --- | --- | --- | --- | --- | --- | --- | --- | --- | --- | --- | --- |
|  | **OR** | **95%-CI** | | **p-value** | **OR** | **95%-CI** | | **p-value** | **OR** | **95%-CI** | | **p-value** |
|  |  | **Low.** | **Up.** |  |  | **Low.** | **Up.** |  |  | **Low.** | **Up.** |  |
| Gender |  |  |  |  | N.S. |  |  |  |  |  |  |  |
| Women (ref) | - | - | - | - |  |  |  |  | - | - | - | - |
| Men | .707 | .635 | .787 | <.001 |  |  |  |  | .830 | .738 | .933 | .002 |
| Age in categories |  |  |  |  |  |  |  |  |  |  |  |  |
| 18-24 years | 1.360 | 1.048 | 1.766 | .021 | 1.540 | 1.166 | 2.036 | .002 | 1.810 | 1.371 | 2.390 | <.001 |
| 25-34 years | 1.617 | 1.416 | 1.846 | <.001 | 1.425 | 1.237 | 1.640 | <.001 | 1.495 | 1.294 | 1.726 | <.001 |
| 35-44 years | 1.165 | 1.038 | 1.308 | .010 | 1.102 | .973 | 1.247 | .126 | 1.270 | 1.120 | 1.441 | <.001 |
| 45-54 years (ref) | - | - | - | - | - | - | - | - | - | - | - | - |
| 55-64 years | .710 | .630 | .800 | <.001 | .773 | .681 | .879 | <.001 | .729 | .637 | .835 | <.001 |
| 65-88 years | .565 | .436 | .733 | <.001 | .671 | .509 | .885 | .005 | .788 | .600 | 1.036 | .088 |
| Educational level |  |  |  |  |  |  |  |  |  |  |  |  |
| Low | .781 | .671 | .910 | .001 | .691 | .588 | .812 | <.001 | .635 | .534 | .755 | <.001 |
| Middle | 1.079 | .982 | 1.185 | .113 | .837 | .757 | .927 | <.001 | .777 | .700 | .862 | <.001 |
| High (ref) | - | - | - | - | - | - | - | - | - | - | - | - |
| Comorbidity | N.S. |  |  |  | N.S. |  |  |  |  |  |  |  |
| No (ref) |  |  |  |  |  |  |  |  | - | - | - | - |
| Yes |  |  |  |  |  |  |  |  | .829 | .752 | .913 | <.001 |
| Hospital admission  during acute  COVID-19 infection | N.S. |  |  |  |  |  |  |  | N.S. |  |  |  |
| No (ref) |  |  |  |  | - | - | - | - |  |  |  |  |
| Yes |  |  |  |  | .657 | .545 | .791 | <.001 |  |  |  |  |
| Time since infection |  |  |  |  |  |  |  |  |  |  |  |  |
| ≤6 months | .783 | .682 | .900 | <.001 | 1.124 | .969 | 1.304 | .121 | .458 | .391 | .536 | <.001 |
| 7-12 months | .846 | .752 | .952 | .006 | .933 | .823 | 1.057 | .273 | .676 | .596 | .766 | <.001 |
| 13-18 months | .970 | .865 | 1.087 | .601 | .750 | .664 | .848 | <.001 | .856 | .759 | .965 | .011 |
| >18 months (ref) | - | - | - | - | - | - | - | - | - | - | - | - |
| Severe fatigue |  |  |  |  |  |  |  |  |  |  |  |  |
| No | .811 | .689 | .954 | .012 | .819 | .688 | .975 | .025 | .713 | .595 | .854 | <.001 |
| Yes (ref) | - | - | - | - | - | - | - | - | - | - | - | - |
| Dyspnoea |  |  |  |  |  |  |  |  | N.S. |  |  |  |
| Grade 1 (ref) | - | - | - | - | - | - | - | - |  |  |  |  |
| Grade 2 | 1.186 | 1.060 | 1.327 | .003 | 1.000 | .886 | 1.128 | .995 |  |  |  |  |
| Grade 3 | 1.321 | 1.179 | 1.480 | <.001 | 1.133 | 1.005 | 1.277 | .041 |  |  |  |  |
| Grade 4 | 1.365 | 1.087 | 1.712 | .007 | 1.225 | .968 | 1.550 | .091 |  |  |  |  |
| Grade 5 | 1.202 | .932 | 1.550 | .156 | 1.515 | 1.175 | 1.955 | .001 |  |  |  |  |
| Cognitive problems |  |  |  |  |  |  |  |  |  |  |  |  |
| None | .629 | .516 | .766 | <.001 | .905 | .741 | 1.104 | .325 | .679 | .548 | .842 | <.001 |
| Slight | .917 | .821 | 1.025 | .126 | .864 | .766 | .975 | .018 | .853 | .755 | .964 | .011 |
| Moderate (ref) | - | - | - | - | - | - | - | - | - | - | - | - |
| Severe | 1.045 | .937 | 1.167 | .427 | 1.137 | 1.013 | 1.277 | .029 | 1.026 | .910 | 1.156 | .677 |
| Extreme | .972 | .784 | 1.206 | .797 | 1.556 | 1.255 | 1.930 | <.001 | 1.316 | 1.054 | 1.644 | .015 |
| Likely anxiety  disorder |  |  |  |  |  |  |  |  | N.S. |  |  |  |
| No (ref) | - | - | - | - | - | - | - | - |  |  |  |  |
| Yes | 1.389 | 1.245 | 1.550 | <.001 | 1.293 | 1.163 | 1.437 | <.001 |  |  |  |  |
| Likely major  depressive disorder |  |  |  |  | N.S. |  |  |  |  |  |  |  |
| No (ref) | - | - | - | - |  |  |  |  | - | - | - | - |
| Yes | 1.188 | 1.068 | 1.322 | .002 |  |  |  |  | 1.225 | 1.102 | 1.362 | <.001 |
| General practitioner |  |  |  |  | N.S. |  |  |  |  |  |  |  |
| No | .755 | .606 | .940 | .012 |  |  |  |  | .682 | .523 | .889 | .005 |
| Yes (ref) | - | - | - | - |  |  |  |  | - | - | - | - |
| Paramedic |  |  |  |  | N.S. |  |  |  |  |  |  |  |
| No | 1.433 | 1.205 | 1.705 | <.001 |  |  |  |  | .723 | .580 | .902 | .004 |
| Yes (ref) | - | - | - | - |  |  |  |  | - | - | - | - |
| Medical specialist |  |  |  |  |  |  |  |  | N.S. |  |  |  |
| No | 1.129 | 1.025 | 1.243 | .014 | .550 | .494 | .613 | <.001 |  |  |  |  |
| Yes (ref) | - | - | - | - | - | - | - | - |  |  |  |  |
| Occupational physician | N.S. |  |  |  |  |  |  |  | N.S. |  |  |  |
| No |  |  |  |  | .874 | .780 | .979 | .020 |  |  |  |  |
| Yes (ref) |  |  |  |  | - | - | - | - |  |  |  |  |
| Mental health  professional | N.S. |  |  |  |  |  |  |  |  |  |  |  |
| No |  |  |  |  | .595 | .541 | .655 | <.001 | .733 | .665 | .808 | <.001 |
| Yes (ref) |  |  |  |  | - | - | - | - | - | - | - | - |

A total of 10,413 respondents were included in regression analyses, due to missing data on gender or educational level (n=49). N.S.= not significant (p>0.05).

Barrier 4: I felt uncomfortable asking for help, because I felt like a burden.

Barrier 5: I had to wait a long time until help was available.

Barrier 6: The help I sought was not reimbursed.

**Table 2-C. Multivariable binomial logistic regression analyses per barrier for barriers 7-9.**

|  | Barrier 7 | | | | Barrier 8 | | | | Barrier 9 | | | |
| --- | --- | --- | --- | --- | --- | --- | --- | --- | --- | --- | --- | --- |
|  | **OR** | **95%-CI** | | **p-value** | **OR** | **95%-CI** | | **p-value** | **OR** | **95%-CI** | | **p-value** |
|  |  | **Low.** | **Up.** |  |  | **Low.** | **Up.** |  |  | **Low.** | **Up.** |  |
| Gender |  |  |  |  |  |  |  |  | N.S. |  |  |  |
| Women (ref) | - | - | - | - | - | - | - | - |  |  |  |  |
| Men | 1.445 | 1.267 | 1.648 | <.001 | .830 | .717 | .961 | .013 |  |  |  |  |
| Age in categories |  |  |  |  |  |  |  |  |  |  |  |  |
| 18-24 years | 1.946 | 1.394 | 2.716 | <.001 | 1.900 | 1.356 | 2.663 | <.001 | 1.947 | 1.359 | 2.789 | <.001 |
| 25-34 years | 1.388 | 1.162 | 1.659 | <.001 | 1.575 | 1.314 | 1.888 | <.001 | 1.524 | 1.248 | 1.862 | <.001 |
| 35-44 years | 1.159 | .989 | 1.359 | .069 | 1.272 | 1.083 | 1.495 | .003 | 1.282 | 1.069 | 1.539 | .008 |
| 45-54 years (ref) | - | - | - | - | - | - | - | - | - | - | - | - |
| 55-64 years | .853 | .725 | 1.005 | .057 | .908 | .769 | 1.071 | .251 | .825 | .678 | 1.004 | .055 |
| 65-88 years | .851 | .624 | 1.159 | .305 | .785 | .564 | 1.092 | .151 | .861 | .592 | 1.252 | .433 |
| Educational level |  |  |  |  |  |  |  |  |  |  |  |  |
| Low | .463 | .372 | .575 | <.001 | .595 | .477 | .742 | <.001 | .675 | .531 | .858 | .001 |
| Middle | .556 | .487 | .635 | <.001 | .838 | .736 | .953 | .007 | .708 | .608 | .824 | <.001 |
| High (ref) | - | - | - | - | - | - | - | - | - | - | - | - |
| Comorbidity | N.S. |  |  |  | N.S. |  |  |  | N.S. |  |  |  |
| No (ref) |  |  |  |  |  |  |  |  |  |  |  |  |
| Yes |  |  |  |  |  |  |  |  |  |  |  |  |
| Hospital admission  during acute  COVID-19 infection |  |  |  |  | N.S. |  |  |  | N.S. |  |  |  |
| No (ref) | - | - | - | - |  |  |  |  |  |  |  |  |
| Yes | .632 | .495 | .807 | <.001 |  |  |  |  |  |  |  |  |
| Time since infection |  |  |  |  |  |  |  |  | N.S. |  |  |  |
| ≤6 months | .335 | .271 | .416 | <.001 | .333 | .267 | .414 | <.001 |  |  |  |  |
| 7-12 months | .497 | .424 | .582 | <.001 | .419 | .354 | .495 | <.001 |  |  |  |  |
| 13-18 months | .638 | .552 | .736 | <.001 | .658 | .570 | .760 | <.001 |  |  |  |  |
| >18 months (ref) | - | - | - | - | - | - | - | - |  |  |  |  |
| Severe fatigue | N.S. |  |  |  |  |  |  |  | N.S. |  |  |  |
| No |  |  |  |  | .772 | .615 | .969 | .026 |  |  |  |  |
| Yes (ref) |  |  |  |  | - | - | - | - |  |  |  |  |
| Dyspnoea | N.S. |  |  |  |  |  |  |  |  |  |  |  |
| Grade 1 (ref) |  |  |  |  | - | - | - | - | - | - | - | - |
| Grade 2 |  |  |  |  | 1.107 | .946 | 1.295 | .205 | 1.050 | .877 | 1.257 | .596 |
| Grade 3 |  |  |  |  | 1.216 | 1.039 | 1.423 | .015 | 1.350 | 1.138 | 1.602 | <.001 |
| Grade 4 |  |  |  |  | 1.269 | .938 | 1.716 | .122 | 1.832 | 1.344 | 2.496 | <.001 |
| Grade 5 |  |  |  |  | 1.790 | 1.318 | 2.431 | <.001 | 1.786 | 1.269 | 2.514 | <.001 |
| Cognitive problems |  |  |  |  |  |  |  |  | N.S. |  |  |  |
| None | 1.200 | .956 | 1.505 | .116 | .904 | .702 | 1.164 | .434 |  |  |  |  |
| Slight | 1.006 | .864 | 1.171 | .938 | .941 | .808 | 1.095 | .430 |  |  |  |  |
| Moderate (ref) | - | - | - | - | - | - | - | - |  |  |  |  |
| Severe | 1.323 | 1.139 | 1.535 | <.001 | .965 | .828 | 1.124 | .645 |  |  |  |  |
| Extreme | 1.707 | 1.313 | 2.218 | <.001 | 1.490 | 1.147 | 1.935 | .003 |  |  |  |  |
| Likely anxiety  disorder |  |  |  |  | N.S. |  |  |  |  |  |  |  |
| No (ref) | - | - | - | - |  |  |  |  | - | - | - | - |
| Yes | 1.260 | 1.101 | 1.441 | <.001 |  |  |  |  | 1.492 | 1.288 | 1.727 | <.001 |
| Likely major  depressive disorder | N.S. |  |  |  | N.S. |  |  |  | N.S. |  |  |  |
| No (ref) |  |  |  |  |  |  |  |  |  |  |  |  |
| Yes |  |  |  |  |  |  |  |  |  |  |  |  |
| General practitioner |  |  |  |  | N.S. |  |  |  | N.S. |  |  |  |
| No | .626 | .442 | .886 | .008 |  |  |  |  |  |  |  |  |
| Yes (ref) | - | - | - | - |  |  |  |  |  |  |  |  |
| Paramedic |  |  |  |  | N.S. |  |  |  | N.S. |  |  |  |
| No | 1.296 | 1.028 | 1.635 | .028 |  |  |  |  |  |  |  |  |
| Yes (ref) | - | - | - | - |  |  |  |  |  |  |  |  |
| Medical specialist |  |  |  |  |  |  |  |  |  |  |  |  |
| No | .642 | .559 | .738 | <.001 | .738 | .641 | .849 | <.001 | .806 | .697 | .933 | .004 |
| Yes (ref) | - | - | - | - | - | - | - | - | - | - | - | - |
| Occupational physician |  |  |  |  |  |  |  |  | N.S. |  |  |  |
| No | 1.206 | 1.050 | 1.385 | .008 | 1.253 | 1.092 | 1.437 | .001 |  |  |  |  |
| Yes (ref) | - | - | - | - | - | - | - | - |  |  |  |  |
| Mental health  professional |  |  |  |  |  |  |  |  |  |  |  |  |
| No | .877 | .776 | .990 | .034 | .762 | .674 | .862 | <.001 | .859 | .749 | .986 | .030 |
| Yes (ref) | - | - | - | - | - | - | - | - | - | - | - | - |

A total of 10,413 respondents were included in regression analyses, due to missing data on gender or educational level (n=49). N.S.= not significant (p>0.05).

Barrier 7: No help was available for my specific needs.

Barrier 8: According to the care provider/organization, I was not eligible for help.

Barrier 9: The person I asked for help didn’t have time.

**Table 2-D. Multivariable binomial logistic regression analyses per barrier for barriers 10-11.**

|  | Barrier 10 | | | | Barrier 11 | | | |
| --- | --- | --- | --- | --- | --- | --- | --- | --- |
|  | **OR** | **95%-CI** | | **p-value** | **OR** | **95%-CI** | | **p-value** |
|  |  | **Low.** | **Up.** |  |  | **Low.** | **Up.** |  |
| Gender |  |  |  |  | N.S. |  |  |  |
| Women (ref) | - | - | - | - |  |  |  |  |
| Men | .707 | .580 | .861 | <.001 |  |  |  |  |
| Age in categories |  |  |  |  |  |  |  |  |
| 18-24 years | 2.417 | 1.676 | 3.484 | <.001 | 2.135 | 1.342 | 3.394 | .001 |
| 25-34 years | 2.063 | 1.669 | 2.551 | <.001 | 1.600 | 1.206 | 2.123 | .001 |
| 35-44 years | 1.425 | 1.165 | 1.743 | <.001 | 1.456 | 1.135 | 1.869 | .003 |
| 45-54 years (ref) | - | - | - | - | - | - | - | - |
| 55-64 years | .659 | .520 | .834 | <.001 | .959 | .745 | 1.236 | .748 |
| 65-88 years | .598 | .356 | 1.003 | .051 | .908 | .567 | 1.453 | .686 |
| Educational level | N.S. |  |  |  | N.S. |  |  |  |
| Low |  |  |  |  |  |  |  |  |
| Middle |  |  |  |  |  |  |  |  |
| High (ref) |  |  |  |  |  |  |  |  |
| Comorbidity | N.S. |  |  |  | N.S. |  |  |  |
| No (ref) |  |  |  |  |  |  |  |  |
| Yes |  |  |  |  |  |  |  |  |
| Hospital admission  during acute  COVID-19 infection | N.S. |  |  |  | N.S. |  |  |  |
| No (ref) |  |  |  |  | - | - | - | - |
| Yes |  |  |  |  | 1.396 | 1.051 | 1.854 | .021 |
| Time since infection |  |  |  |  |  |  |  |  |
| ≤6 months | .761 | .592 | .977 | .032 | .348 | .244 | .496 | <.001 |
| 7-12 months | .864 | .706 | 1.059 | .159 | .479 | .372 | .618 | <.001 |
| 13-18 months | .836 | .686 | 1.018 | .075 | .710 | .572 | .882 | .002 |
| >18 months (ref) | - | - | - | - | - | - | - | - |
| Severe fatigue | N.S. |  |  |  | N.S. |  |  |  |
| No |  |  |  |  |  |  |  |  |
| Yes (ref) |  |  |  |  |  |  |  |  |
| Dyspnoea |  |  |  |  |  |  |  |  |
| Grade 1 (ref) | - | - | - | - | - | - | - | - |
| Grade 2 | 1.306 | 1.069 | 1.595 | .009 | 1.136 | .874 | 1.477 | .340 |
| Grade 3 | 1.230 | 1.007 | 1.503 | .043 | 1.610 | 1.261 | 2.056 | <.001 |
| Grade 4 | 1.470 | 1.026 | 2.105 | .036 | 2.606 | 1.798 | 3.776 | <.001 |
| Grade 5 | 1.263 | .842 | 1.894 | .260 | 2.713 | 1.823 | 4.039 | <.001 |
| Cognitive problems |  |  |  |  |  |  |  |  |
| None | .515 | .347 | .765 | .001 | .629 | .393 | 1.004 | .052 |
| Slight | .593 | .477 | .737 | <.001 | .958 | .749 | 1.224 | .730 |
| Moderate (ref) | - | - | - | - | - | - | - | - |
| Severe | 1.124 | .940 | 1.344 | .200 | 1.361 | 1.091 | 1.697 | .006 |
| Extreme | 1.393 | 1.025 | 1.892 | .034 | 1.587 | 1.102 | 2.286 | .013 |
| Likely anxiety  disorder | N.S. |  |  |  |  |  |  |  |
| No (ref) |  |  |  |  | - | - | - | - |
| Yes |  |  |  |  | 1.365 | 1.121 | 1.661 | .002 |
| Likely major  depressive disorder |  |  |  |  | N.S. |  |  |  |
| No (ref) | - | - | - | - |  |  |  |  |
| Yes | 1.430 | 1.219 | 1.678 | <.001 |  |  |  |  |
| General practitioner | N.S. |  |  |  | N.S. |  |  |  |
| No |  |  |  |  |  |  |  |  |
| Yes (ref) |  |  |  |  |  |  |  |  |
| Paramedic | N.S. |  |  |  | N.S. |  |  |  |
| No |  |  |  |  |  |  |  |  |
| Yes (ref) |  |  |  |  |  |  |  |  |
| Medical specialist |  |  |  |  |  |  |  |  |
| No | .783 | .657 | .931 | .006 | .657 | .522 | .828 | <.001 |
| Yes (ref) | - | - | - | - | - | - | - | - |
| Occupational physician |  |  |  |  |  |  |  |  |
| No | 1.278 | 1.072 | 1.523 | .006 | 1.550 | 1.266 | 1.897 | <.001 |
| Yes (ref) | - | - | - | - | - | - | - | - |
| Mental health  professional |  |  |  |  |  |  |  |  |
| No | .781 | .667 | .913 | .002 | .716 | .592 | .867 | <.001 |
| Yes (ref) | - | - | - | - | - | - | - | - |

A total of 10,413 respondents were included in regression analyses, due to missing data on gender or educational level (n=49). N.S.= not significant (p>0.05).

Barrier 10: The help or aid I wanted was too expensive.

Barrier 11: It was difficult to apply for help due to complicated laws and regulations.
