# Supplementary material for: Self-perceived barriers to healthcare access for patients with post COVID-19 condition
Source: BMC Health Serv Res. 2024 Sep 6;24:1035. doi: 10.1186/s12913-024-11488-w (PMC11378429; doi:10.1186/s12913-024-11488-w)
Supplement: Supplementary file 2 — Supplementary Material 2. [file 12913_2024_11488_MOESM2_ESM.docx]

# **Additional File 2**

**General information about the Dutch healthcare system**

- The health insurance system in the Netherlands combines public and private insurance. All residents are required to purchase basic health insurance, which covers essential medical services. This basic insurance is purchased from private insurers, who are required to accept all applicants. Adults also pay an annual deductible (€385 in 2024). Additional health insurance is optional. The Dutch Healthcare Authority (NZa) is responsible for the quality and affordability of healthcare services, specifies essential services and determines maximum tariffs for services.
- General practitioners (GPs) serve as gatekeepers in the Dutch healthcare system. Consultations with GPs and preventative services are free and the annual deductible does not apply. GPs manage patient referrals to specialists and hospitals, but patients can often choose the hospital or specialist they are referred to.
- Long-term care for chronic conditions is largely funded through a public insurance schema (the Long-Term Care Act).

**Care for PCC within the Dutch healthcare system**

- Between July 2020 and July 2024, a temporary paramedical recovery care scheme was available for PCC patients, through which patients had access to a physiotherapist, occupational therapist, speech therapist and dietitian. Patients were referred through a GP or medical specialist within six months after the acute phase of the disease. The maximum duration of recovery care was six months with a possibility of an additional six month period based on assessment by a GP or medical specialist.
- In addition, several hospitals set up PCC clinics to assess and treat patients with long-term complaints.

**The role of C-support**

- C-support is a Dutch foundation, commissioned by the Dutch Ministry of Health, that supports and provides advice to individuals who experience long-term symptoms after COVID-19. Patients can self-register at C-support through an online form when they experience these long-term complaints. C-support then assesses the support needs of patients, helps with finding appropriate healthcare professionals, and works with experts (e.g., labour experts) to address PCC-related problems in different areas. They do not provide medical care. In addition, they share knowledge with healthcare professionals.

**References**

- Wammes J, Stadhouders N, Westert G. International Health Care System Profiles-Netherlands. The Commonwealth Fund. June 5, 2020.
- Zorginstituut Nederland. Paramedische herstelzorg na COVID-19. Available from: <https://www.zorginstituutnederland.nl/Verzekerde+zorg/paramedische-herstelzorg-na-covid-19> [Accessed June 28, 2024].
- C-support. About C-support. Available from: <https://www.c-support.nu/en/about-c-support/> [Accessed June 28, 2024].
